# Ballistic Performance, Thermal and Chemical Characterization of Ubim Fiber (*Geonoma baculifera*) Reinforced Epoxy Matrix Composites

**DOI:** 10.3390/polym15153220

**Published:** 2023-07-28

**Authors:** Belayne Zanini Marchi, Pedro Henrique Poubel Mendonça da Silveira, Wendell Bruno Almeida Bezerra, Lucio Fabio Cassiano Nascimento, Felipe Perissé Duarte Lopes, Verônica Scarpini Candido, Alisson Clay Rios da Silva, Sergio Neves Monteiro

**Affiliations:** 1 Military Institute of Engineering, IME, Praça General Tibúrcio 80, Urca, Rio de Janeiro 22290-270, Brazil; pedroo.poubel@gmail.com (P.H.P.M.d.S.); wendellbez@gmail.com (W.B.A.B.); lucio@ime.eb.br (L.F.C.N.); snevesmonteiro@gmail.com (S.N.M.); 2 Laboratory for Advanced Materials—LAMAV, State University of North Fluminense, UENF, Campos dos Goytacazes 28013-602, RJ, Brazil; perisse@uenf.br; 3 Materials Science and Engineering, Federal University of Para, UFPA, Highway BR-316, km 7.5–9.0, Ananindeua 67000-000, Brazil; scarpini@ufpa.br (V.S.C.); alissonrios@ufpa.br (A.C.R.d.S.)

**Keywords:** ubim fiber, epoxy composite, ballistic behavior, thermal stability, DSC, TGA, FTIR

## Abstract

The search for unexplored natural materials as an alternative to synthetic components has driven the development of novel polymeric composites reinforced with environmentally-friendly materials. Natural lignocellulosic fibers (NLFs) have been highlighted as potential reinforcement in composite materials for engineering applications. In this work, a less known Amazonian fiber, the ubim fiber (*Geonoma baculifera*), is investigated as a possible reinforcement in epoxy composites and was, for the first time, thermally characterized by thermogravimetric analysis (TGA) and differential scanning calorimetry (DSC). Additionally, its chemical structure was elucidated by Fourier transform infrared spectroscopy (FTIR). Ballistic tests were also performed against the threat of a 7.62 mm high-speed lead projectile. The results were statistically analyzed by the Weibull statistical analysis method. FTIR analysis showed the functional groups normally found for NLFs highly rich in cellulose, hemicellulose, and lignin. The TGA/DTG results showed the onset of thermal degradation for the composites (325~335 °C), which represents better thermal stability than isolated ubim fiber (259 °C), but slightly lower than that of pure epoxy (352 °C). The DSC results of the composites indicate endothermic peaks between 54 and 56 °C, and for the ubim fibers, at 71 °C. Ballistic tests revealed higher energy absorption in composites with lower fiber content due to the more intense action of the brittle fracture mechanisms of the epoxy resin, which tended to dissipate more energy. These failure mechanisms revealed the presence of river marks, cracks, and broken fibers with a detachment interface. These results may contribute to the production of ubim fiber-reinforced composites in engineering applications, such as ballistic armors.

## 1. Introduction

Environmental regulations have stimulated research in several areas related to sustainability. International organizations concerned with the negative impacts of human activity on the environment are seeking sustainable solutions, and the reduction of environmental impact has become a priority for governments, companies, and researchers around the world. Currently, there is a growing trend toward using natural lignocellulosic fibers (NLFs) as substitutes for synthetic fibers. This practice is gaining relevance due to their potential to offer an ecologically correct alternative. NLFs are obtained from renewable sources and are biodegradable, lightweight, and low-cost, making them a viable option in sectors such as fabrics, plastics, and construction. Additionally, the use of NLFs reduces dependence on oil-based materials and helps to mitigate the environmental impact associated with the production of synthetic fibers. This trend aims to create a more balanced and environmentally conscious future [1,2,3,4].

**Figure 1:**
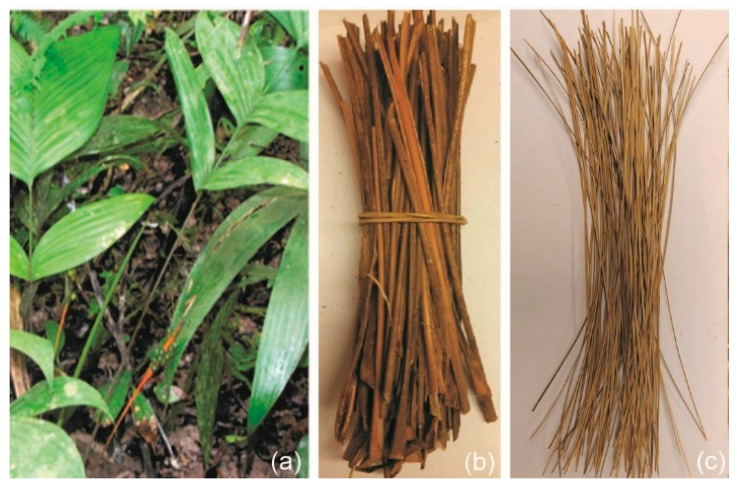
(**a**) Ubim plant; (**b**) Plant stems collected in Belém do Pará, Brazil; (**c**) Fiber extracted from the stem.

The use of (NLFs) is increasingly common as reinforcement for polymeric matrix composites [3,4,5,6,7,8], as they have been shown to exhibit mechanical properties similar to or even greater than composites produced with synthetic fibers [9,10]. Studies conducted with various types of NLFs have reported their success as reinforcement in polymeric composites for engineering applications [11], including in the automotive industry [12], building construction [13], and ballistic armor [14,15,16,17]. Among the NLFs with potential application as reinforcement in polymer matrix composites, the fiber extracted from the ubim, a less-known plant, stands out. The ubim is a plant of the palm family, scientifically known as *Geonoma baculifera*, of multiple stems, smooth, with elongated and unbranched fibers [18]. Figure 1a illustrates the ubim plant, and Figure 1b illustrates the ubim stems collected in Belém, Brazil, along with the fiber extracted from the stem, observed in Figure 1c.

Ubim fiber is typically found in forests with high rainfall. There are occurrence data in Guyana, Peru, Bolivia, and Venezuela [18]. In Brazil, it can be found in the states of Amazonas, Amapá, Pará, Maranhão, and Piauí. The ubim is one of the fibrous plant species that plays a prominent role in the daily life of traditional Amazonian communities since its leaves are used in hut roofs, awnings, and boat linings, while the stem is used in fish traps [19]. However, there are no reports about the thermal and chemical properties of ubim fiber-reinforced composites to establish technical limits for possible engineering applications.

Thermosetting composites are commonly manufactured using epoxy resins due to their low cost. Epoxy resins are used to fabricate composites reinforced with natural fibers because of their low cost, ease of fabrication, and good properties [20]. Typically, these composites are produced with synthetic fibers to improve mechanical properties. However, synthetic fibers are not environmentally friendly and have a non-renewable origin. In contrast, natural fibers are readily available in nature, biodegradable, low-cost, and come from renewable sources [21]. Nevertheless, natural fibers exhibit the undesirable characteristic of incompatibility with the polymer matrix due to the cellulose constituents present in the fibers, which can lead, in certain cases, to inferior mechanical properties. Despite these characteristics, the investigation of new natural fibers as reinforcements in engineering and ballistic applications is valid from a sustainable standpoint [22]. It is possible, through the definition of specific applications, to mitigate the environmental effects caused by polymers by using eco-friendly materials.

The objective of this paper is to conduct the first investigation of the ballistic performance of epoxy matrix composites reinforced with ubim fiber. Additionally, the complementary study included thermal and chemical characterization of these composites using techniques such as Fourier-transform infrared spectroscopy (FTIR), thermogravimetric analysis (TGA), and differential scanning calorimetry (DSC). These techniques provided unprecedented data on the thermal and chemical properties of the material under study.

## 2. Materials and Methods

### 2.1. Materials

The ubim stems illustrated in Figure 1a were collected in the region of Belém, state of Pará, Brazil. The commercial epoxy resin used was the bisphenol A diglycidyl ether (DGEBA) epoxy, and triethylenetetramine (TETA) was selected as the hardener in a stoichiometric ratio of 100:13. Both components are manufactured by Dow Chemical, (São Paulo, Brazil), and distributed by Resin-poxy Ltd. (Rio de Janeiro, Brazil).

### 2.2. Composites Preparation

**Figure 2:**
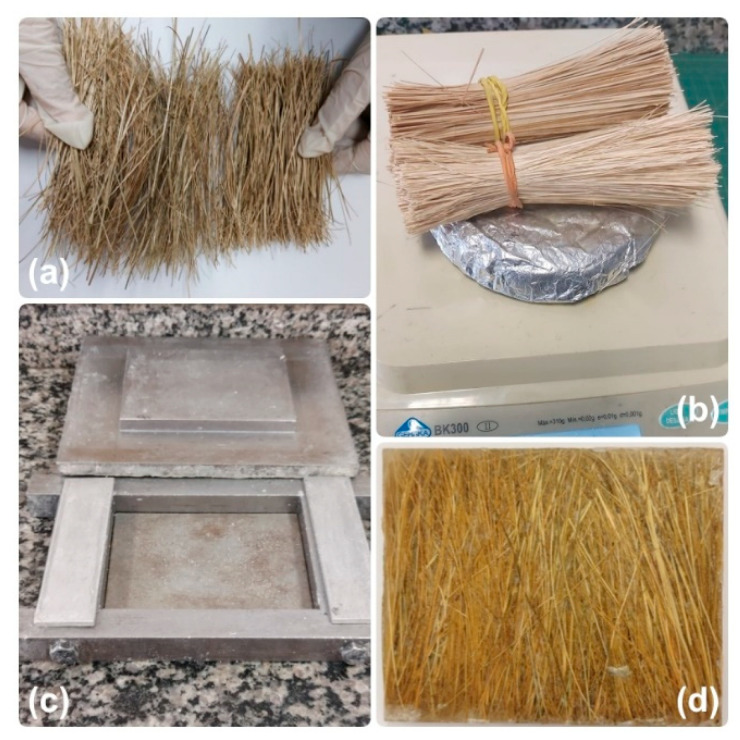
(**a**) Ubim fibers cut to a length of 15 cm; (**b**) Ubim fibers weighed to obtain the correct volume fraction to reinforce the composites; (**c**) Mold used in the preparation of composites; (**d**) Composite plate after compression process.

The ubim fibers were received in the form of stems, as shown in Figure 1a. The fibers were immersed in water for 24 h to remove impurities, followed by drying in an oven at 70 °C for 24 h. The fibers were separated and cut to a length of 150 mm and weighed, Figure 2a,b, according to the volume of fibers to be inserted into a rectangular mold of 150 mm × 120 mm × 12 mm to manufacture the composite plates, Figure 2c,d.

The ubim fibers were unidirectionally positioned in the mold, Figure 2c, in proportions of 10, 20, and 30 vol.% and incorporated with the epoxy-hardener mixture DGEBA/TETA still liquid in the proper proportions. The processing of composites was carried out by compression, generally used in recent works [23,24,25]. The plates were pressed under a load of 5 tons in a hydraulic press SKAY (São Paulo, Brazil) for 24 h, after which the mold was opened, and the plates were removed, as shown in Figure 2d.

### 2.3. Characterization

#### 2.3.1. Fourier Transform Infrared Spectroscopy (FTIR)

FTIR analysis of ubim fibers, epoxy, and composites was performed on a Spectrum 100 FT-IR Spectrometer from Perkin Elmer (São Paulo, Brazil), equipped with an attenuated total reflectance (ATR) detector. The samples were ground to the finest powder possible to perform the test. The analyses were carried out with scanning in a spectral range from 4000 to 650 cm^−1^, 60 scans with a resolution of 4 cm^−1^. The generated data were treated with the Origin Pro software, generating the respective transmittance spectra (%) as a function of the wavenumber (cm^−1^).

#### 2.3.2. Thermogravimetric Analysis (TGA)

The thermogravimetric analysis (TGA) of ubim fibers, epoxy, and composites were performed with a thermogravimetric analyzer from TA Instruments, model TGA Q-500 (New Castle, DE, USA). The samples were manually crushed with a pestle/mortar and placed in a platinum crucible. The TGA and DTG curves were obtained, at room temperature, up to 700 °C, under a nitrogen atmosphere, with a heating rate of 10 °C/min, as specified by ASTM E1131 [26].

#### 2.3.3. Differential Scanning Calorimetry (DSC)

The differential scanning analysis (DSC) of ubim fiber and composites were performed on a Shimadzu DSC-60 machine (Tokyo, Japan) under a nitrogen atmosphere with a heating rate of 10 °C/min and a temperature range of 20 to 400 °C for all samples.

#### 2.3.4. Ballistic Tests

The ballistic tests were conducted at the Army Assessment Center (CAEx) in the Marambaia peninsula, Rio de Janeiro, Brazil. All tests were carried out according to the NIJ 0101.06 [27] standard using 7.62 × 51 mm caliber ammunition with 9.7 g of mass.

The projectile was shot from a gun barrel and hit the composite plate, positioned 15 m from the gun, with a perpendicular (90°) trajectory. The optical barrier with the HPI B471 chronograph and model Weibel SL-520P Doppler radar measured both the impact velocity ($v_{i}$) against the target plate and the residual velocity ($v_{r}$) of the projectile leaving the plate after perforation. Fractured samples of each component after the ballistic test were analyzed by scanning electron microscopy (SEM) in a model QUANTA FEG250 Fei microscope operating with secondary electrons at 15 kV.

The absorbed ballistic impact energy ($E_{abs}$) of the target plate is given by Equation (1) [28]:

(1) $$E_{abs}=\frac{m\left(v_{i}{}^{2}-v_{r}{}^{2}\right)}{2}$$

where m is the mass, $v_{i}$ is the impact velocity, and $v_{r}$ is the residual velocity.

Another important parameter is the limit velocity (*V_L_*), which can be estimated by manipulating Equation (1) when the kinetic energy of the projectile is completely absorbed and the residual velocity is zero. This can be expressed mathematically as in Equation (2):

(2) $$V_{L}=\sqrt{\frac{2E_{abs}}{m}}$$

**Figure 3:**
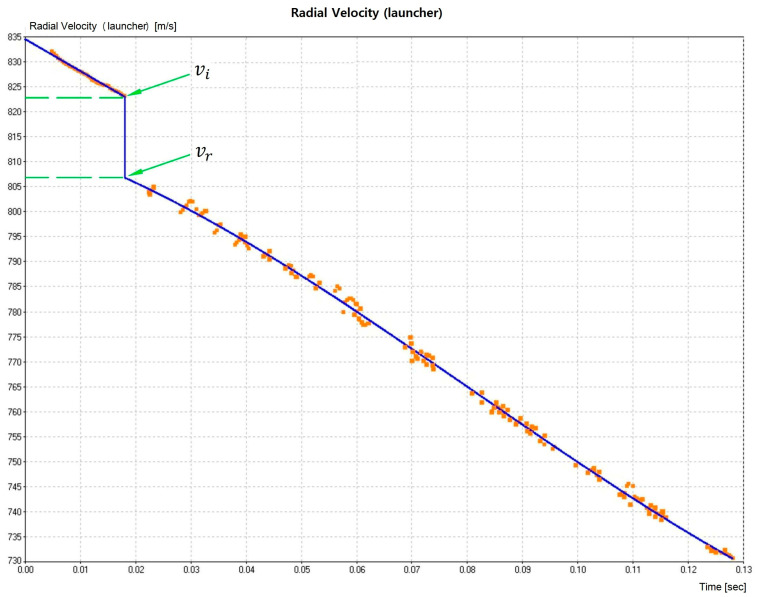
Experimental points obtained from the radar spectrum and the FFT curve fitting.

The data captured by the radar provides a frequency spectrum over time, for which the intensity and velocity can be correlated using Fast Fourier Transform (FFT) to obtain the velocity curve fitting shown in Figure 3. This figure illustrates the experimental points obtained for a composite sample of 30 vol.% ubim fiber, along with the adjusted continuous polynomial curve. Additionally, it should be noted that a sudden drop occurs at ~823 m/s, indicating the velocity at the instant of impact. After this point, the velocity decreases to ~807 m/s, illustrating the residual velocity.

#### 2.3.5. Weibull Statistical Analysis

The absorbed energy values for each investigated composite were statistically evaluated using Weibull Analysis (WA). A logarithm-based linear expression [29] allows the graphical interpretation of the Weibull parameters:

(3) $$\ln\left[\ln\left(\frac{1}{F\left(x\right)}\right)\right]=\beta \ln x-\left(\beta \ln\theta \right)$$

where x is the absorbed energy, β is the Weibull modulus, and θ is the characteristic energy.

## 3. Results and Discussion

### 3.1. FTIR Results

**Figure 4:**
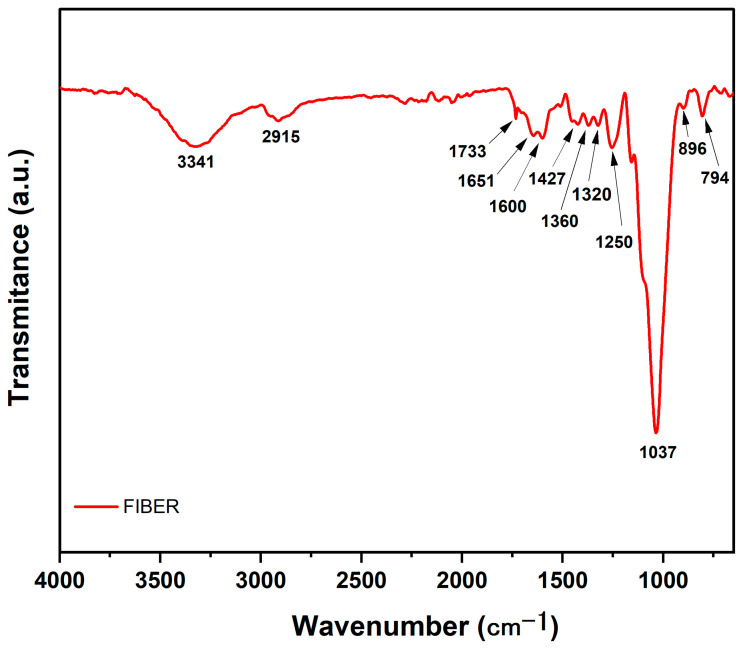
FTIR spectra of ubim fiber.

**Figure 5:**
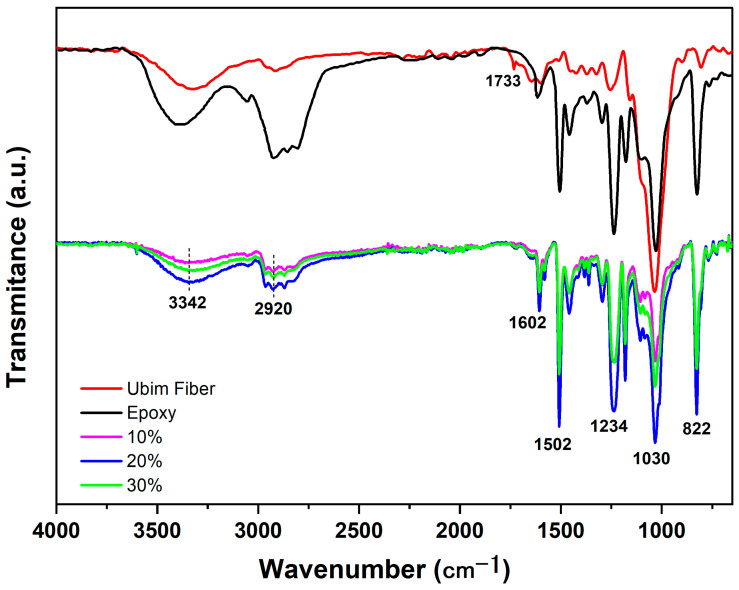
The FTIR spectra of composites incorporated with 10, 20, and 30 vol.% ubim fiber and plain epoxy for comparison.

The FTIR analyses of both the individual ubim fiber in Figure 4 and related composites in Figure 5 were performed to characterize the functional groups participating in chemical interactions with ubim fiber constituents.

Figure 4 shows the FTIR spectra of ubim fiber in association with absorption bands of characteristic chemical groups. A relatively wide band centered at a 3341 cm^−1^ peak is typical of (O-H) (hydroxyl) associated with water at the NLFs’ surface [30,31]. The narrower band at 2915 cm^−1^ is another typically reported in NLFs and attributed to (C-H) stretching of aliphatic-rich groups in the cellulose and hemicellulose [32,33,34]. The band at 1733 cm^−1^ corresponds to the (C=O) bonding of carboxylic acid, aliphatic, and ketone functional groups onto lignin [35,36]. Another (C=O) band at 1651 cm^−1^ might be assigned to aromatic rings in the hemicellulose [17]. The band at 1600 cm^−1^ corresponds to the (C=C) aromatic ring in the lignin [37]. While the band at 1427 cm^−1^ is reported to be associated with (CH_2_) groups of the cellulose [34,38]. The two small peaks at 1360 and 1320 cm^−1^ have been attributed to the stretching vibration of the (CH_2_) group and symmetrical angular deformation on the (CH_2_) functional group [39]. As for the 1250 cm^−1^ band, it is recognized as the (C-O) stretching of the lignin acetyl functional group [40,41]. The highest intensity band at 1037 cm^−1^ is well-known for NLFs in association with (C-O) functional groups of cellulose [34]. The next band at 896 cm^−1^ appears from the (C-O-C) stretching related to the B-glycosidic bonding in cellulose and hemicellulose [42]. Finally, the 794 cm^−1^ band is considered characteristic of the lignin and assigned to the aromatic group (C-H) stretching [43].

Figure 5 shows the FTIR spectra of epoxy composites incorporated with 10, 20, and 30 vol.% ubim fiber jointly with individual ubim fiber and plain epoxy for comparison.

The composite bands match those of both the ubim fiber and the plain epoxy with small displacements from the bands described for the individual ubim fiber in Figure 4. The epoxy bands and corresponding functional groups reported in the literature [44,45] are presented in Table 1.

The FTIR bands listed in Table 1 for the DGEBA/TETA epoxy used as a matrix for the present composites are also shown in Figure 5. An additional explanation is given for these main bands. Between 3500 and 3400 cm^−1^ are the typical (O-H) stretching of the surface moisture [48]. The 3055 cm^−1^ band corresponds to the oxirane (C-H) stretching. The range from 2965 to 2873 cm^−1^ is attributed to the stretching of the (C-H) and (CH_2_) groups from aromatic and aliphatic polymeric chains [45,49]. Less intense bands at 1610 and 1509 cm^−1^ can be assigned to stretching of (C=C) and (C-C) aromatic groups, respectively [49,50]. As for the 1241 cm^−1^ band, Jiang et al. [51] indicated it to be associated with the vibrational mode of the (C-O) aromatic carbon group. The final bands from 1035 to 830 cm^−1^ are attributed by Sengül and Tüzün [52] to (C-O-C) stretching of ethers of the epoxy oxirane group.

A comparison between the composites and DGEBA/TETA epoxy FTIR spectra in Figure 5 reveals similar bands as expected due to the matrix predominance. While comparing the composites spectra in Figure 5 with the spectrum of individual ubim fiber in Figure 4, the 3341, 2915, 1600, and 1037 cm^−1^ bands are similar. This is a clear indication of functional group interaction between ubim fibers and epoxy matrix.

It is also worth mentioning that the 1733 cm^−1^ band for the ubim in Figure 5, attributed to (C=O) binding of carboxylic and aliphatic and ketone groups in the lignin [36,53], is not visible in the epoxy spectrum. This might be justified by the fact that these groups are characteristic of NLFs and not synthetic polymers [34,53].

### 3.2. Thermogravimetric Analysis (TGA)

**Figure 6:**
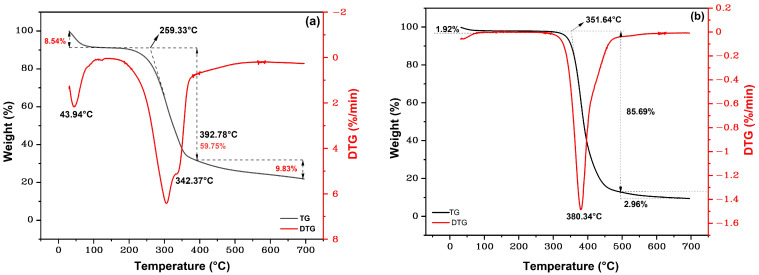
TGA and DTG curves for (**a**) The ubim fibers; (**b**) The neat epoxy.

Figure 6 shows the TGA/DTG curves for (a) the ubim fiber and (b) the neat epoxy. Beyond 700 °C, the TG curve remained horizontally constant, indicating no further mass loss.

The TGA/DTG curve of the ubim fiber in Figure 6a comprises information on the thermal events along the test. In the initial stage of thermal degradation, from 25 to 200 °C, a mass reduction of 8.54% was observed. This is attributed to the loss of fiber surface moisture, which is expected for hydrophilic NFLs [54]. In the second stage, the most significant degradation occurs between 200 and 400 °C, associated with a mass loss of 59.75%. This can be assigned to the thermal decomposition of NLFs statement components such as hemicellulose, cellulose, and lignin that degrade when exposed above 220 °C [17,55,56]. The corresponding DTG curve in Figure 4a indicates that this second event displays a main peak at 305 °C, which might be associated with the degradation of both hemicellulose and cellulose glycosidic [57]. Another shoulder peak at 342.37 °C can be related to the decomposition of cellulose I and α-cellulose [58,59]. The investigation reported similar peaks for hemp (308.2 °C), jute (298.2 °C), and kenaf (307.2 °C) [60]. Above 400 °C, a third stage of thermal degradation occurs at a slower rate until 700 °C in association with a mass loss of 9.83%, corresponding to the decomposition of lignin.

**Figure 7:**
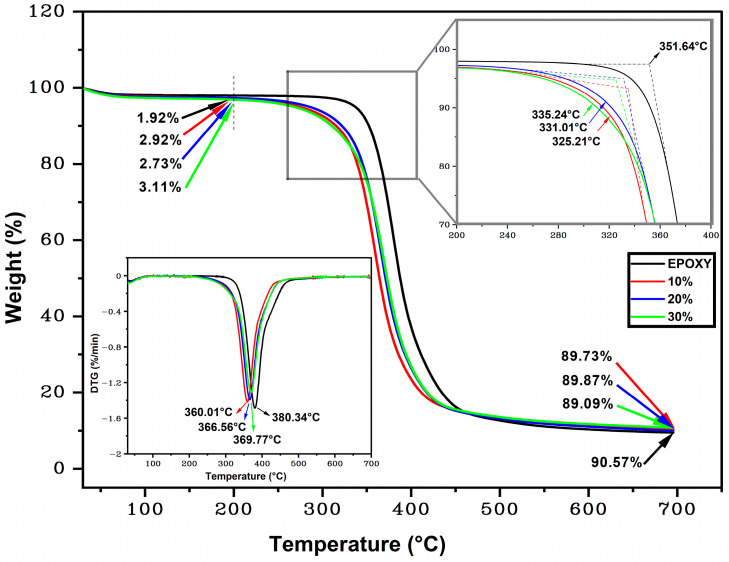
TGA and DTG curves of composites and epoxy.

Figure 6b shows the TGA/DTG curves for the plain DGEBA/TETA epoxy used as a composite matrix. Three stages of mass loss are also identified. These are well-reported in the literature [46]. In Figure 6b, it is possible to observe the first stage of degradation that occurs between room temperature (25 °C) and 300 °C. In this initial phase, there is a slight mass loss due to the removal of moisture present in the epoxy, in addition to water vapor generated during heating [47]. A mass loss of 1.92% is recorded at 200 °C, a value that remains stable until the end of the first stage of degradation. The second stage of degradation begins near T_onset_. At around 350 °C, the second stage that extends up to 500 °C with the highest mass loss of 85.69% associated with thermal degradation of the epoxy polymeric chains is initiated [61]. Finally, in the third degradation stage, the epoxy carbonization corresponds to a mass loss of 2.96% up to 700 °C. Similar results were reported by Junio et al. [62]. Figure 7 shows the TGA/DTG curves for ubim fiber reinforced epoxy composites with respective changes for 10, 20, and 30 vol.% fiber contents.

These changes are presented in Table 2 for the corresponding three stages.

By comparing the results in Figure 6a and Figure 7 as well in Table 2, one finds that the composites disclose improved thermal stability than the isolated ubim fiber. Indeed, the onset of the 10, 20, and 30 vol.% ubim fiber composites’ thermal degradation takes place at higher temperatures (325.21, 331.01, and 335.24 °C, respectively) than the isolated ubim fiber (259.33 °C).

Additionally, a displacement of the maximum decomposition of cellulose temperature peak is noticed from 360.01 to 369.77 °C as the fiber content increases from 10 to 30 vol.%, respectively. This fact might be related to several factors, such as the composite thermal stability with respect to the ubim fiber or to the epoxy resin [63]. In general, the onset temperature for thermal degradation of polymer composites reinforced with NLF is around 200 °C [55,63,64]. The present work corroborates this value, as shown in Figure 7. It is of relevance for applications requiring working temperatures up to 200 °C for which the investigated ubim fiber composites can be considered thermally stable.

### 3.3. Differential Scanning Calorimetry

**Figure 8:**
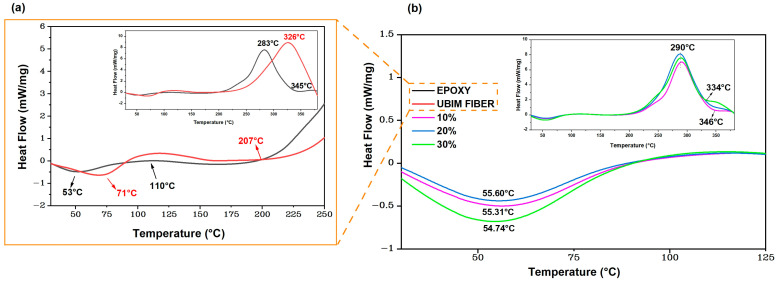
Differential scanning calorimetry (DSC) curves for (**a**) Epoxy resin and ubim fibers; (**b**) Composites 10–30 vol.%.

Figure 8 shows the DSC curves obtained for the neat epoxy and the ubim fiber, as well as the epoxy/ubim composites. The lower transition peak on the DSC plot reveals the endothermic (heat required) region, while the upward transition represents the exothermic (heat released) region.

The ubim fibers DSC thermogram in Figure 8a displays an initial endothermic peak at 71 °C, which is attributed to the release of moisture naturally adhered to the surface of a hydrophilic fiber. These results confirm the finding of the ubim fiber TG curve in Figure 6a of humidity loss before 100 °C. Other NLFs disclose similar endothermic peaks as 64 °C in caranan [65], 76 °C in titica vine [66], 80 °C in kenaf [61], 88 °C in palm rachis [67], and 61 °C in jute fiber [68].

In Figure 8a, a DSC exothermic peak is also noted at 320 °C for the ubim fiber, which can be assigned to the maximum thermal degradation of cellulose and hemicellulose [59] beginning around 207 °C.

As for the plain epoxy, Figure 8a reveals an initial endothermic peak at 53 °C, which is associated with the glass transition temperature (T_g_) [69]. This temperature corresponds to a polymeric state modification towards higher macromolecule chain mobility [70]. In Figure 8a, a subtle exothermic peak is also observed around 110 °C, which corresponds to an additional epoxy cure reaction [69]. This behavior was reported in studies related to the cure kinetic of NLFs epoxy composites [62,63]. Another exothermic event for the plain epoxy in Figure 6a is noticed at 283 °C, which might be attributed to crosslinks occurring in the resin homopolymerization and esterification [66]. Finally, a possible exothermic event at 345 °C for the epoxy in Figure 6a is associated with the onset of intense mass loss by thermal degradation depicted in the TG curve of Figure 6b due to the rupture of epoxy chains [61,66]. Regarding the composites in Figure 8b, the DSC curves exhibit quite similar behavior for all investigated ubim fiber contents (10 to 30 vol.%). The initial Tg endothermic peak discloses a small increase in temperature (54.74 to 55.31 °C) compared with the plain epoxy (53 °C). These peaks may also be assigned to the release of moisture in the ubim fibers surface, which explains the comparative increase in temperature with fiber content [55,62,63]. Exothermic peaks from 280 to 290 °C in the composites inset of Figure 8b, as well as the plain epoxy peak in Figure 8a, are probably a consequence of crosslinks reactions (homopolymerization and esterification) of the epoxy groups [66]. Furthermore, the maximum rate of hemicellulose decomposition occurs in this range of temperatures. In Figure 8b, a last exothermic event is detected between 334 and 346 °C, which could be associated not only to rupture of epoxy chains, like in Figure 4b, but also to possible thermal decomposition of lignin in the ubim fiber [61,66]. The aforementioned DSC results in Figure 8 corroborate those presented for TGA in Figure 6 and revealed the thermal limitation of epoxy composites reinforced with ubim fibers.

### 3.4. Ballistic Tests

**Figure 9:**
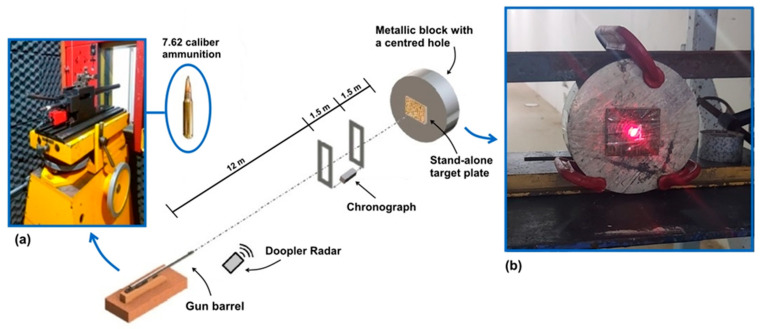
Schematic diagram of the CAEX shooting line showing, in accordance with the NIJ standard procedure [27]: (**a**) The gun barrel and the 7.62 caliber ammunition used; and (**b**) The measurement procedure using a laser beam indicating the direction of the 7.62 mm, projectile with fixation plate attached to a round metal block.

Figure 9 illustrates the schematic for a typical ballistic test using a 7.62 mm (Figure 8a) projectile against ubim fabric-reinforced epoxy composites. The sample was fixed onto a metallic block with a 50 mm diameter, preparing it for the ballistic test. The projectile was positioned, aided by a laser sight, ensuring that it was fired directly at the desired target on the sample, as shown in Figure 9b.

Table 3 presents the results obtained for average impact velocity (*v_i_*), average residual velocity (*v_r_*), absorbed energy (*E_abs_*), and the estimated limit velocity (*V_L_*).

The values of absorbed energy were used to employ the WA in order to determine the characteristics and reliability trends of the samples ranging from 0–30 vol.% of fibers, as presented in Table 4.

**Figure 10:**
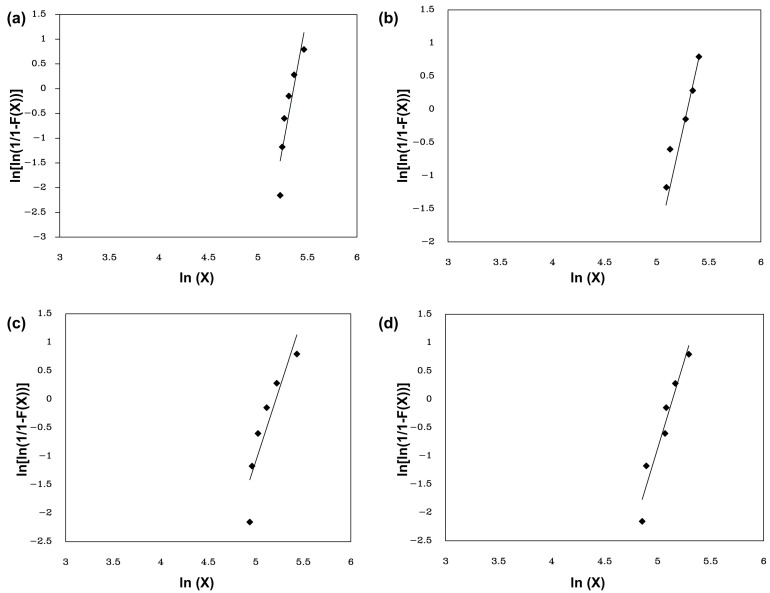
Weibull plots for the absorbed energy of epoxy composites with (**a**) Neat epoxy; (**b**) 10 vol%; (**c**) 20 vol%; and (**d**) 30 vol% ubim fiber.

Figure 10 shows the Weibull plots of absorbed energy for the investigated epoxy composites.

Based on the values of the R^2^ statistical measure, it was observed that the linear model was adequately adjusted to the data since all the tested groups had coefficients greater than 0.82, indicating a high statistical representativeness of the distribution. The closer the value of R^2^ is to 1, the better the fit and the smaller the associated error. Furthermore, it should be noted that the characteristic value of the Weibull parameter θ is similar to the average absorbed energy.

**Figure 11:**
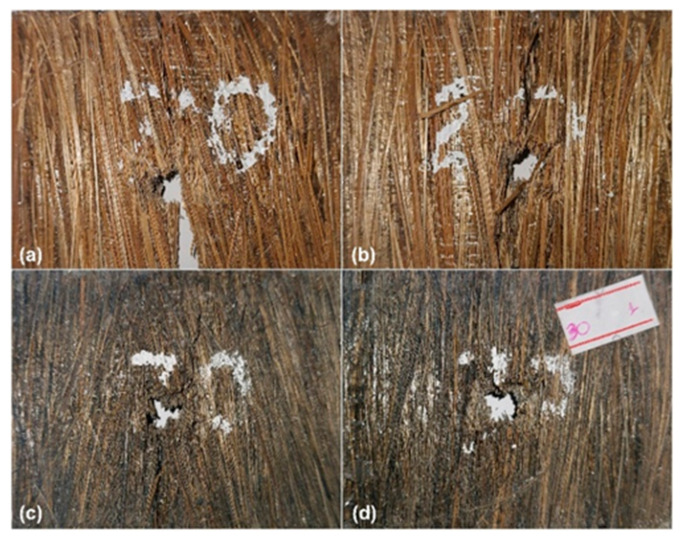
Visual aspect of the composite target after the ballistic impact for ubim fiber reinforced epoxy composites with (**a**,**b**) 20 vol.% fiber; (**c**,**d**) 30 vol.% fiber.

The physical integrity of the composite plates (CPs) is also an important factor to consider when using materials for ballistic armor. In the residual velocity experiments conducted with 7.62 mm ammunition, all samples were perforated by the projectile. The tested samples are shown in Figure 11.

Figure 11 shows the specimens’ appearance after the residual velocity test. The plates containing 10 vol.% of fibers, as well as the epoxy resin plate, fragmented into several pieces after the ballistic impact, making photographic documentation impossible. In the plates with 20 vol.% of ubim fibers, represented by Figure 11a,b, the fragmentation of the samples is also observed, with the loss of some pieces and, in some cases, the plate splitting into two parts. This behavior is a significant and undesirable problem in materials intended for ballistic purposes.

**Figure 12:**
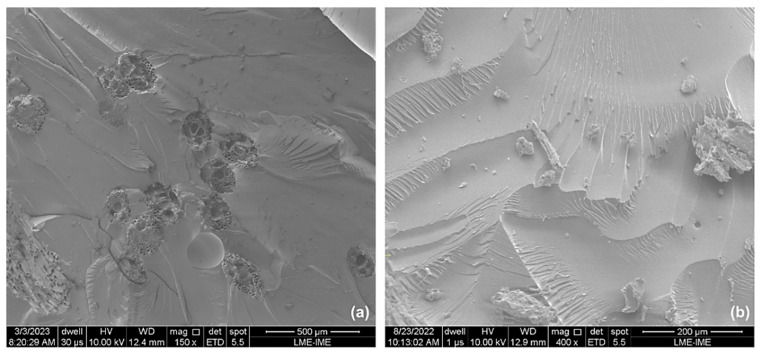
SEM micrographs of the ballistic impact region: (**a**) 10 vol% with magnification of 150×; (**b**) 10 vol% with magnification of 400×.

**Figure 13:**
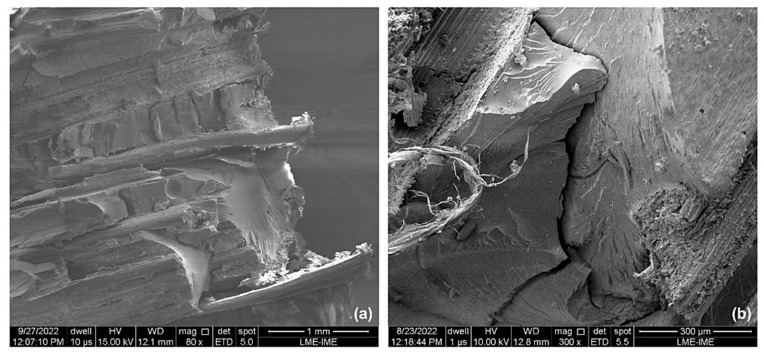
SEM micrographs of the ballistic impact region: (**a**) 20 vol% with magnification of 80×; (**b**) 20 vol% with magnification of 300×.

**Figure 14:**
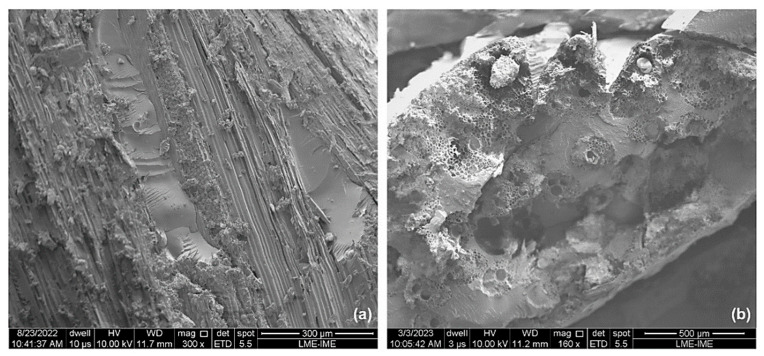
SEM micrographs of the ballistic impact region: (**a**) 30 vol% with magnification of 300×; (**b**) 30 vol% with magnification of 160×.

Samples with 30 vol.% presented better physical integrity than all previous samples, although they had less energy absorption capacity after impact. However, not all samples of this formulation showed this phenomenon, as some also had cracks but with interrupted propagation due to the superior performance of the ubim fibers. The importance of physical integrity in test specimens of materials intended for personal ballistic armor is highlighted in some previous studies [16,71,72]. In order to obtain a more detailed evaluation of the fracture mechanisms, micrographs were taken using scanning electron microscopy. These micrographs helped identify the fracture mechanisms in both the fibers and the matrix. The images were obtained for composites containing 10 to 30 vol.% of ubim fibers and are illustrated in Figure 12, Figure 13 and Figure 14.

Figure 12a,b, which depict the composite with 10 vol.% of ubim fibers, reveal several active failure mechanisms. The fracture surface shows the presence of cracks and broken fibers with a detachment interface, indicating weak adhesion between the fiber and the epoxy matrix of the composite. This weak adhesion is a limiting factor for the increase in strength and rigidity [73]. In addition, the presence of river marks in the matrix is also evident, which is associated with typical failure that occurs in natural fibers/polymer resin in a region with a higher resin concentration [74].

It is noteworthy that the composites with 10 vol.% ubim fibers presented relatively higher values of absorbed energy compared to the other composites. This characteristic may be attributed to the more intense action of the brittle fracture mechanisms of the epoxy resin, which is evidenced by the strong presence of river marks. Since river marks are associated with many surface fractures after impact, they tend to dissipate more energy. The absorption of kinetic energy by the reinforcement phase was also evidenced by the appearance of fiber ruptures and cracks on the material surface, which has also been reported by Luz et al. [72] and Assis et al. [75].

Figure 13 displays fracture surfaces that reveal several failure mechanisms, including river marks, imperfections, broken fibers, cracks, fibril separation, matrix fracture, and delamination. Delamination is a mechanism that occurs when the interfacial adhesion between the matrix and the fiber is impaired [16]. In this case, the different nature of the hydrophobic polymeric matrix and the hydrophilic fiber results in layer separation. Poor interfacial interaction may have been responsible for the early failure and low values of absorbed energy.

Figure 14 reveals some repeated failure mechanisms, such as fiber breakage, matrix fracture, detachment, and delamination, as seen in the composites with 10 and 20 vol.% ubim fibers. Additionally, voids formed on the surface of the resin due to fiber pullout can be observed. The cross-section of the fibers also shows the presence of lumens and cell walls. As previously mentioned, delamination negatively impacts the integrity of the composites. However, the samples with 30 vol.% of ubim fibers demonstrated better dimensional stability after ballistic impact compared to the previous samples. Despite presenting a lower value of absorbed energy, the performance of the fibers was quite evident in the images.

The ballistic resistance of epoxy composites reinforced with ubim fibers against 7.62 mm caliber ammunition is comparable to that of other composites reinforced with natural fibers and/or renewable natural components. Table 5 presents a comparison of the ballistic resistance of various composites reinforced with natural fibers based on the energy absorbed during a 7.62 mm ammunition test.

When comparing experimental data with the results from different studies published under similar conditions, it is noticeable that ubim fiber-reinforced composites demonstrate ballistic performance equivalent to several other natural fiber-reinforced composites. Specifically, the composite containing 10% of the volume of ubim fibers shows ballistic performance close to that of epoxy matrix composites reinforced with Piassava fibers [16], PALF fibers [71], and fique fabric [77].

On the other hand, composites reinforced with guaruman fibers [78], hemp fibers [25], and arapaima scales [76] exhibited inferior ballistic performance when compared to epoxy/ubim composites.

It is relevant to mention that the work published by Reis et al. [78] found that even with lower absorbed energy, the composites still met the normative standards for ballistic tests, reinforcing the viability of using natural fibers in ballistic applications. Thus, it is concluded that the use of natural fibers in ballistic applications is a viable and promising alternative.

## 4. Summary and Conclusions

In the present work, the thermal and chemical behavior of ubim fiber reinforced epoxy matrix composites (*Geonoma baculifera*) were, for the first time, investigated using the techniques of thermogravimetry, differential scanning calorimetry, and Fourier transform infrared spectroscopy. Composites with different volumetric fiber fractions were also ballistically tested for the first time with 7.62 mm ammunition. The surfaces of the fractured samples were analyzed by scanning electron microscopy (SEM). Based on the results obtained, the following conclusions were defined:

- The results of the TGA analysis indicated that there was intermediate thermal stability in the composites when compared to the thermal behavior of both neat epoxy resin and ubim fiber. The volumetric fraction of fibers added as reinforcement influenced the thermal stability of the composites. The initial degradation temperature, as well as the maximum degradation temperature, decreased as more fiber was added. On average, the composites had significant mass loss above 325 °C, which may be a working proposition for the limited use of ubim fiber epoxy composites around this temperature.
- The DSC curves revealed a slight increase in the temperature of the endothermic peak of the composites in relation to the peak of the pure epoxy, observed by the displacement of the curves. This difference is related to the amount of fibers added as reinforcement due to the moisture present in the ubim fiber, in addition to a possible contribution of the epoxy matrix to the glass transition temperature (T_g_).
- The T_g_ was obtained at 71 °C for the ubim fiber, at 53 °C for the pure epoxy resin, and ranging from 54.74 to 55.60 °C for the ubim fiber reinforced epoxy composites.
- The FTIR analysis showed expected results, with bands referring to molecular vibrations of functional groups belonging to the basic constituents of natural lignocellulosic fibers (NLFs), such as OH, CH_2_, C-O, and C-O-C, highly rich in cellulose, hemicellulose, and lignin.
- These results emphasize the importance of using NLFs as reinforcement in polymeric composites and add value to the existing knowledge about fibers, especially ubim, which has not yet been published in the literature. This study should contribute to the multiple applications expected for ubim fiber-reinforced epoxy composites in terms of thermal stability and chemical properties.
- The ballistic tests of residual velocity indicated that the energy absorbed by the composite plates decreased as the ubim fiber content increased. The same behavior was observed in the limit velocity measurements.
- The absorbed energy (*E_abs_*) and the Weibull characteristic parameter were found to be quite similar within standard deviations.
- The visible macrocracks developed in the composites containing 10 and 20 vol% of ubim fibers compromise their potential application in ballistic armor. However, in the composite with 30 vol% of fibers, only the projectile’s perforation hole was visible, indicating that its integrity was maintained.
- SEM analysis revealed that the main damage responsible for macrocracks is associated with detachment and delamination of the fiber/matrix, indicating poor adhesion at the interface. Additionally, fiber breakage and river marks were frequently observed.
- The more intense performance of the brittle fracture mechanisms of the epoxy resin tended to dissipate more energy, which may explain the high absorption energy in the composites with a smaller volumetric fraction.
- The ballistic performance of the composites, even though it may have been reduced due to the increase in ubim fiber content, still compares to that of other natural fibers documented in the literature, making it a viable alternative for use in ballistic armor.

## Figures and Tables

**Table 1:** Absorption FTIR bands of DGEBA/TETA epoxy [46,47].

Wavenumber (cm^−1^)	Functional Groups
3400	Stretching of (O-H) binding
3055	Stretching of (C-H) and oxirane ring
2965–2873	Stretching of (C-H) and (CH_2_) aromatic and aliphatic groups
1610	Stretching of (C=C) aromatic
1509	Stretching of (C-C) aromatics
1241	Stretching of (C-O) aromatic ring
1035	Stretching of (C-O-C) ethers
830	Stretching of (C-O-C) binding of the oxirane groups

**Table 2:** Thermogravimetric parameters for the neat epoxy, plain fiber, and ubim fiber composites.

Sample	Mass Loss Up to 200 °C (%)	Initial Degradation Temperature (°C)	Temperature of Maximum Degradation Rate (°C)	Mass Loss at the End of Second Stage (%)	Mass Loss at 700 °C (%)
Ubim	8.54	259.33	305.68	68.29	78.12
Epoxy	1.92	351.64	380.34	87.61	90.57
10%	2.92	325.21	360.01	85.44	89.73
20%	2.73	331.01	366.56	85.34	89.87
30%	3.11	335.24	369.77	84.95	89.09

**Table 3:** Parameters obtained in the residual velocity test for the 0–30 vol.% samples.

Samples	$v_{i}\;(m/s)\;$	$v_{r}\;(m/s)\;$	$E_{abs}\;(J)$	$V_{L}\;(m/s)$
Epoxy	812.58 ± 3.84	786.28 ± 5.93	203.82 ± 18.92	204.82 ± 9.36
10 vol.%	833.75 ± 12.81	810.28 ± 15.39	187.03 ± 25.99	195.98 ± 13.57
20 vol.%	808.42 ± 9.84	786.56 ± 10.81	169.07 ± 33.73	185.98 ± 18.00
30 vol.%	815.28 ± 8.70	794.85 ± 10.54	159.42 ± 26.32	180.79 ± 14.93

**Table 4:** Weibull distribution for the energy absorbed after impact with 7.62 mm ammunition in composites with 0–30 vol.% ubim fiber.

Samples	Standard Deviation	β	θ	R^2^
Epoxy	17.27	10.74	212.84	0.83
10%	23.73	7.06	199.19	0.84
20%	30.79	5.13	183.55	0.82
30%	24.03	6.20	170.91	0.93

**Table 5:** Comparison of the absorbed energy of ubim fiber-reinforced composites with other natural fiber-reinforced composites found in the literature.

Composite	*E_abs_* (J)	Reference
10 vol.% ubim fibers	187.03	PW *
20 vol.% ubim fibers	169.07	PW *
30 vol.% ubim fibers	159.42	PW *
Epoxy/Piassava fibers	272	[16]
Epoxy/Arapaima Scales	100	[76]
Epoxy/PALF	212	[71]
Epoxy/Fique	211	[77]
Epoxy/Hemp	108	[25]
Guaruman	105	[78]

* PW—Present Work.

## Data Availability

The data presented in this study are available on request from the corresponding author.

## Sample Availability

Samples of the compounds are available from the authors.
